# Mental Health Disparities Within the LGBT Population: A Comparison Between Transgender and Nontransgender Individuals

**DOI:** 10.1089/trgh.2015.0001

**Published:** 2016-01-01

**Authors:** Dejun Su, Jay A. Irwin, Christopher Fisher, Athena Ramos, Megan Kelley, Diana Ariss Rogel Mendoza, Jason D. Coleman

**Affiliations:** ^1^ Center for Reducing Health Disparities, College of Public Health, University of Nebraska Medical Center, Omaha, Nebraska.; ^2^ Department of Health Promotion, Social and Behavioral Health, College of Public Health, University of Nebraska Medical Center, Omaha, Nebraska.; ^3^ Department of Sociology & Anthropology, University of Nebraska at Omaha, Omaha, Nebraska.; ^4^ Department of Health Promotion and Sexology, School of Public Health, Curtin University, Perth, Australia.; ^5^ Department of Nutrition and Health Services, College of Education and Human Sciences, University of Nebraska-Lincoln, Lincoln, Nebraska.; ^6^ Eastern Nebraska Community Action Partnership (ENCAP), Omaha, Nebraska.; ^7^ School of Health, Physical Education, and Recreation, University of Nebraska at Omaha, Omaha, Nebraska.

**Keywords:** depression symptoms, discrimination, LGBT, LGBT identity acceptance, transgender

## Abstract

**Purpose:** This study assessed within a Midwestern LGBT population whether, and the extent to which, transgender identity was associated with elevated odds of reported discrimination, depression symptoms, and suicide attempts.

**Methods:** Based on survey data collected online from respondents who self-identified as lesbian, gay, bisexual, and/or transgender persons over the age of 19 in Nebraska in 2010, this study performed bivariate *t*- or chi-square tests and multivariate logistic regression analysis to examine differences in reported discrimination, depression symptoms, suicide attempts, and self-acceptance of LGBT identity between 91 transgender and 676 nontransgender respondents.

**Results:** After controlling for the effects of selected confounders, transgender identity was associated with higher odds of reported discrimination (OR=2.63, *p*<0.01), depression symptoms (OR=2.33, *p*<0.05), and attempted suicides (OR=2.59, *p*<0.01) when compared with nontransgender individuals. Self-acceptance of LGBT identity was associated with substantially lower odds of reporting depression symptoms (OR=0.46, *p*<0.001).

**Conclusion:** Relative to nontransgender LGB individuals, transgender individuals were more likely to report discrimination, depression symptoms, and attempted suicides. Lack of self-acceptance of LGBT identity was associated with depression symptoms among transgender individuals.

## Introduction

Transgender is an umbrella term for persons whose gender identity, gender expression, or behavior does not conform to that typically associated with the sex to which they were assigned at birth.^1^ While the lesbian, gay, bisexual, and transgender (LGBT) population overall bears a disproportionate burden of mental health issues relative to the general population,^2–10^ emerging evidence suggests that within the LGBT population the odds of depression symptoms are even higher among transgender individuals compared with nontransgender individuals.^11–15^ Findings based on a major survey conducted in 2009 by MassEquality revealed that heterosexual respondents had the lowest rates of depression and suicide ideation, followed by gay and lesbian respondents, bisexual respondents, and transgender respondents, of whom 30.8% had contemplated suicide.^15^ The corresponding prevalence of suicide ideation was even higher in the National Transgender Discrimination Survey (NTDS) where 41% of transgender respondents reported ever attempting suicide in their life compared with 1.6% among the general population in the United States.^16^

The elevated odds of suicide attempt among transgender persons could be related to discrimination experienced by this population. Discrimination is an important risk factor of depression among transgender individuals.^17^ Clements-Nolle, Marx, and Katz^18^ found depression and gender-based discrimination to be independently associated with attempted suicide in their San Francisco-based study, in which 32% of transgender participants had attempted suicide. According to data from NTDS, 63% of transgender individuals had experienced a serious act of discrimination, and nearly a quarter (23%) had experienced three or more serious acts of discrimination, a level of discrimination which the study described as catastrophic.^16^ Transgender discrimination is a common experience across the United States in areas of public life, including housing, employment, accommodation, the workplace, and healthcare settings.^19–21^ Nearly one in five (19%) respondents in the NTDS reported being refused medical care due to their gender identity, and half reported that they had to teach their healthcare providers about transgender care. Over one in four (28%) had postponed healthcare due to discrimination, and nearly half (48%) had postponed healthcare due to affordability. The study found that the transgender population had lower income than the general population and nearly four times more likely to have a household income of under $10,000/year.^16^

In a heteronormative society, nontraditional gender identity and sexuality influence self-esteem. Internalized transphobia, a discomfort with one's own transgenderism as a result of internalizing society's normative gender expectations, can negatively affect health outcomes among transgender people.^22^ For many transgender individuals, feelings of shame, isolation, anger, sadness, loss, and even self-rejection or requestioning of one's identity can lead to depression symptoms, which in turn might be associated with high odds of sexual behavior.^23^ On the other hand, identity acceptance and coping strategies can be protective factors in the face of discrimination,^24^ but when positive reinforcement and social support are lacking, the ability to deal with discrimination and prejudice can be significantly impaired.^25^

This study describes the experience of a sample of gender and sexual minority adults in Nebraska. The study has two aims: the first is to assess whether transgender identity is associated with an elevated probability of reported discrimination, depression symptoms, and suicide attempts compared with nontransgender LGB individuals; the second is to determine if LGBT identity acceptance is associated with a lower probability of depression symptoms in transgender and nontransgender LGB individuals.

## Methods

### Data

The data utilized in the current study were collected in 2010 through an online survey for ease of recruitment over the large geographical area of Nebraska. The survey was approved by the Institutional Review Board at the University of Nebraska Medical Center in 2010. Before the survey, the study team engaged a number of community members and organizations from across the state to better understand the needs of the LGBT community and how research might be helpful in supporting those needs. The survey instruments were developed based on feedback and suggestions from interviewed community members using a community-based participatory research (CBPR) approach.^26,27^ Participants were self-identified lesbian, gay, bisexual, and/or transgender persons over the age of 19. Through a multipronged recruitment strategy, involving e-mails, advertisements in LGBT-friendly publications and fliers at public venues, and nonincentivized respondent-driven sampling, 770 LGBT Nebraskans participated in the study.^28^

Participants were directed to a university-hosted website to complete the survey, where an introductory page informed them of the aims of the study. Participants were informed of their rights on the website and agreed to participate if they met the study criteria (living, working, or accessing services in Nebraska, being 19 years or older, and self-identifying as LGBT). The 67-item survey took on average 30 min to complete and participants could choose to not answer any question about which they felt uncomfortable by selecting “Prefer not to answer.” Participants were given the option to receive a $5 gift card on completion of the survey. Upon opting to receive the gift card, participants were taken to a separate page not linked to the survey to provide their mailing address. Only about half of participants chose to collect the incentive.

### Measures

This analysis includes transgender identity, basic sociodemographic information, basic health indicators, and measures of discrimination, depression symptoms, suicide attempts, and LGBT identity acceptance. Transgender identity was based on the following question: Do you identify as transgender/transsexual or gender nonconforming? (binary; yes vs. no) Sociodemographic and economic status variables included age (a continuous variable ranging from 19 to 70 years), race (binary; white vs. nonwhite), rural (binary; rural vs. urban), marital status (binary; married or partnered vs. all other marital status categories), education (binary; no college degree vs. college degree or higher), income (binary; annual household income <$25,000 vs. annual household income ≥$25,000), and employment status (binary; employed for wages vs. unemployed). Rural residence was defined as residential locations other than the metro areas of Omaha and Lincoln, the two major cities in Nebraska.

The following health indicators were included in this analysis: self-rated health (binary; excellent/very good vs. good/fair/poor), HIV infection status (binary; positive vs. negative or prefer not to answer), smoking daily (binary; yes vs. no or prefer not to answer), and current use of illicit drugs (binary; yes vs. no or prefer not to answer).

Discrimination was measured using a 15-item scale that assessed the frequency of reported discrimination, which was initially developed by Wright et al. in their study of sexual minority youths in Indiana.^29^ Items included questions such as “Were you treated unfairly by employers, bosses, and supervisors because of your LGBT status?” and “Did someone verbally insult or abuse you?” (the frequency was coded as never=1, once=2, twice=3, and thrice or more=4). Overall scores were derived by summing all coded responses with a range from 15 to 60 and an average score of 24 among all respondents; reliability was high (α=0.904). Individuals with a discrimination score above 24 were coded as reporting a high level of discrimination.

Depression symptoms were measured utilizing the Centers for Epidemiological Studies–Depression Scale (CES-D).^30^ The 20-item scale asked participants to indicate how often each item had occurred for them in the past week. Statements included the following: “I felt lonely,” I enjoyed life,” and “I was happy.” Options were ranked on a 4-point scale from “rarely or none of the time (less than 1 day)” to “most or all of the time (5–7 days).” Scores were calculated as per standard protocols, ranging from 0 to 60, with high scores indicating greater depressive symptoms. Reliability for the scale in this study was high (alpha=0.929).^30^ Individuals who scored beyond a proposed clinical cutoff value of 16 were coded as 1, indicating more depression symptoms.

Suicide attempts were assessed using a single-item question: Have you ever attempted suicide? (binary; yes vs. no). Self-acceptance of LGBT identity was measured using a scale typically used to measure internalized homophobia adapted from Wright et al.^29^ Instead of measuring internalized homophobia, the scale on self-acceptance of LGBT identity concerns the degree to which study participants rejected negative attitudes about sexual/gender minority identities. The 11-item scale included statements such as “I have a positive attitude about being LGBT” and “I often feel ashamed that I am LGBT.” Participants indicated their agreement on a 5-point Likert scale from strongly disagree to strongly agree. Reliability for the scale was high (alpha=0.805). Possible scores range from 11 to 55, with scores equal or above 44 coded as having a high level of self-acceptance of LGBT identity and scores below 44 as having a low level of self-acceptance of LGBT identity.

### Statistical analyses

We first used *t*- or chi-square tests to assess if there were significant differences between transgender and nontransgender respondents in demographics, socioeconomic status (SES), health behavior, reported discrimination, depression symptoms, LGBT identity acceptance, and attempted suicide. This was followed by a logit model, in which we related perceiving above-average discrimination to transgender identity after controlling for demographics, SES, self-rated health, HIV status, and health behavior. We then ran two additional logit models, in which we examined how transgender identity, reported discrimination, LGBT identity acceptance, and other selected explanatory variables—demographics, SES, self-rated health, HIV status, and health behavior—could help predict the odds of depression symptoms and suicide attempt, respectively. Finally, we conducted a cross-tabulation and chi-square test to assess the association between LGBT identity acceptance and depression symptoms, respectively, among transgender and nontransgender respondents. In all regression analyses, we also presented information on model fitness as indicated by the percentage of cases predicated correctly by the model and Cox and Snell R Square.^31^ Statistical analyses were conducted using SPSS 21.0.

## Results

We first compared responses to selected variables between respondents who identified as transgender and those who identified otherwise (Table 1). Transgender respondents on average had lower socioeconomic status than the rest of the sample. About 40.7% of transgender respondents received a bachelors or higher degree compared with 54.7% of nontransgender respondents (*p*=0.012). Consistent with the gap in educational attainment, transgender respondents were also less likely to be employed for wages than the rest of the sample (63% vs. 73%, *p*=0.035). Another notable difference is that relative to nontransgender respondents in the sample, transgender respondents were less likely to report being HIV positive (*p*=0.032).

**Table 1.** **A Description of the Sample by Transgender Status (*n*=767)**

	Transgender		Nontransgender		T-test or chi-square	Total sample	
Variables	Mean or %	***N***	Mean or %	***N***	p	Mean or %	***N***
Age (mean years)	36.3	89	36.0	665	0.810	36.0	754
Race					0.224		
White	86.8	79	91.9	614		91.3	691
Nonwhite	13.2	12	8.1	54		8.7	66
Residence					0.729		
Urban (Lincoln/Omaha)	90.4	75	89.1	548		89.3	623
Rural (not from Lincoln/Omaha)	9.6	8	10.9	67		10.7	75
Marital status					0.516		
Married or partnered	52.2	47	55.9	377		55.4	424
Other	47.8	43	44.1	298		44.6	341
Education					0.012		
No college degree	59.3	54	36.4	306		47.1	360
College degree or higher	40.7	37	63.6	370		52.9	407
Income					0.090		
Annual Household $ <25,000	38.2	34	29.4	196		32.7	
Annual Household $ ≥25,000	61.8	55	70.6	471			
Employment status					0.035		
Employed for wages	62.6	57	73.2	495		72.0	552
Unemployed	37.4	34	26.8	181		28.0	215
Self-rated health					0.071		
Excellent/very good	54.4	49	64.2	433		63.1	482
Good/fair/poor	45.6	41	35.8	241		36.9	282
HIV infection status					0.032		
Negative or prefer not to answer	100.0	90	95.1	639		95.5	729
Positive	0.0	0	4.9	33		4.5	33
Smoking daily					0.930		
Yes	16.7	15	16.2	109		16.4	124
No or prefer not to answer	83.3	75	83.8	563		83.6	638
Current use of illicit drugs					0.802		
Yes	70.3	64	71.6	484		28.6	220
No or prefer not to answer	29.7	27	28.4	192		71.4	550
Level of discrimination^a^					0.083		
Low	56.0	51	65.3	439		64.2	490
High	44.0	40	34.7	233		35.8	273
Depression symptoms^b^					<0.001		
Fewer	46.2	42	66.6	447		64.2	489
More	53.8	49	33.4	224		35.8	273
Ever attempted suicide					<0.001		
Yes	37.6	32	15.9	106		18.6	138
No	62.4	53	84.1	559		81.4	602
Self-acceptance of LGBT identity^c^					0.107		
Low	53.8	49	44.9	302		45.9	351
High	46.2	42	55.1	371		54.1	413
Total number of cases		91		676			767
Percentage		11.9		88.1			100.0

^a^ Based on a 15-item scale with scores of 0–24 being coded as Low and 25–60 coded as High.

^b^ Based on a 20-item Centers for Epidemiological Studies–Depression scale with scores ≤16 coded as Fewer and scores >16 as More.

^c^ Based on a 11-item scale with scores ≥44 coded as having a high level of self-acceptance of LGBT identity and scores <44 as having a low level of self-acceptance of LGBT identity.

The most significant differences between transgender and nontransgender respondents, however, were found in past week depressive symptoms and a life history of attempted suicide. Transgender respondents were more likely to report depression symptoms (53.9% vs. 33.4%, *p*<0.001) in the past week, and the proportion of transgender respondents who reported attempted suicide was over twice that of nontransgender respondents (37.7% and 15.9%, respectively, *p*<0.001).

We next assessed the relationship between our selected explanatory variables and perception of above-average discrimination in the sample (Table 2). Transgender respondents were more likely than nontransgender respondents to report high levels of reported discrimination. After controlling for the effects of age, race, SES, self-rated health, and other selected variables, the odds for transgender respondents to report above-average discrimination were more than twice the odds for nontransgender respondents (*p*<0.01). Smoking on a daily basis was associated with higher odds of perceiving above-average discrimination (OR=1.73, *p*<0.05).

**Table 2.** **Multivariate Logistic Regression on Reporting a High Level of Discrimination in the Sample Expressed as Odds Ratios**

Explanatory variables	Odds ratio	95% Confidence interval
Transgender identity		
Nontransgender	1.00	
Transgender	2.09^a^	(1.28, 3.42)
Age	1.01	(1.00, 1.03)
Race		
Nonwhite	1.00	
White	0.82	(0.48, 1.41)
Residence		
Urban (Lincoln/Omaha)	1.00	
Rural (not from Lincoln/Omaha)	0.82	(0.47, 1.43)
Marital status		
Other	1.00	
Married or partnered	1.20	(0.86, 1.69)
Education		
No college degree	1.00	
College degree or higher	1.18	(0.82, 1.69)
Income		
Annual household $ ≥25,000	1.00	
Annual household $ <25,000	1.01	(0.68, 1.50)
Employment status		
Unemployed	1.00	
Employed for wages	1.01	(0.68, 1.50)
Self-rated health		
Good/fair/poor	1.00	
Excellent/very good	0.92	(0.65, 1.29)
HIV infection status		
Negative or prefer not to answer	1.00	
Positive	1.59	(0.75, 3.38)
Smoking daily		
No or prefer not to answer	1.00	
Yes	1.73^b^	(1.11, 2.70)
Current use of illicit drugs		
No or prefer not to answer	1.00	
Yes	1.45	(1.00, 2.10)
Self-acceptance of LGBT identity		
Low	1.00	
High	1.04	(0.75, 1.45)
Model Information		
Number of cases included	670	
Percentage predicted correctly	65.4	
Cox and Snell R Square	0.05	

^a^ *p*<0.01.

^b^ *p*<0.05.

Transgender identity was associated with higher odds of depression symptoms and suicide attempt (Table 3). After controlling for selected variables in our analysis, the odds for transgender respondents to report high levels of depression symptoms were 84% higher than nontransgender respondents (*p*<0.05). Similar findings can also be observed in the case of attempting to commit suicide (OR=2.68, *p*<0.01).

**Table 3.** **Multivariate Logistic Regression on Mental Health Expressed as Odds Ratios**

	More depression symptoms		Ever attempted suicide	
Explanatory variables	Odds ratio	95% CI	Odds ratio	95% CI
Transgender identity				
Nontransgender	1.00		1.00	
Transgender	1.84^a^	[1.06, 1.17]	2.68^b^	(1.31, 5.13)
Age	0.99	[0.97, 1.00]	0.99	(0.97, 1.01)
Race				
Nonwhite	1.00		1.00	
White	1.15	[0.64, 2.07]	0.84	(0.44, 1.62)
Residence				
Urban (Lincoln/Omaha)	1.00		1.00	
Rural (Not from Lincoln/Omaha)	1.19	[0.67, 2.10]	0.96	(0.47, 1.94)
Marital status				
Other	1.00		1.00	
Married or partnered	0.59^c^	[0.41, 0.86]	0.91	(0.58, 1.41)
Education				
No college degree	1.00		1.00	
College degree or higher	0.77	[0.52, 1.14]	0.69	(0.43, 1.10)
Income				
Annual household $ ≥25,000	1.00		1.00	
Annual household $ <25,000	1.86^c^	[1.24, 2.79]	1.64^a^	(1.02, 2.66)
Employment status				
Unemployed	1.00		1.00	
Employed for wages	0.68	[0.45, 1.03]	0.60^a^	(0.37, 0.96)
Self-rated health				
Good/fair/poor	1.00		1.00	
Excellent/very good	0.31^b^	[0.21, 0.45]	0.69	(0.45, 1.08)
HIV infection status				
Negative or prefer not to answer	1.00		1.00	
Positive	1.27	[0.56, 2.89]	2.26	(0.95, 5.41)
Smoking daily				
No or prefer not to answer	1.00		1.00	
Yes	0.75	[0.45, 1.24]	1.19	(0.69, 2.08)
Current use of illicit drugs				
No or prefer not to answer	1.00		1.00	
Yes	1.62^a^	[1.08, 2.44]	0.93	(0.57, 1.51)
Reported discrimination				
Low	1.00		1.00	
High	2.59^b^	[1.77, 3.77]	3.17^b^	(2.05, 4.91)
Self-acceptance of LGBT identity				
Low	1.00		1.00	
High	0.45^b^	[0.31, 0.65]	1.20	(0.77, 1.86)
Model Information				
Number of cases included	669		655	
Percentage predicted correctly	72.9		82.7	
Cox and Snell R Square	0.20		0.11	

^a^ *p*<0.05.

^b^ *p*<0.001.

^c^ *p*<0.01.

Besides transgender identity, several of our selected explanatory variables were also associated with the odds of depression symptoms. Respondents who were married or partnered at the time of the survey were less likely to report high depression symptoms compared with the rest of the sample (OR=0.59, *p*<0.01). Having an annual household income of less than $25,000 was associated with higher odds of depression symptoms (OR=1.86, *p*<0.01). Excellent or very good self-rated health was associated with lower odds of depression symptoms (OR=0.31, *p*<0.001). Respondents who were currently using illicit drugs at the time of the survey were more likely to report depression symptoms (OR=1.62, *p*<0.05).

The two variables that showed the most significant association with depression symptoms were reported discrimination and LGBT identity acceptance. Respondents who perceived above-average discrimination were more likely to report depression symptoms (OR=2.59, *p*<0.001). LGBT identity acceptance was negatively associated with depression symptoms. Respondents with higher identity acceptance had significantly lower odds of reporting depression symptoms compared with those with low identity acceptance (OR=0.45, *p*<0.001).

In terms of the odds of attempted suicide, having a household income of less than $25,000 was associated with higher odds of attempt to commit suicide (OR=1.64, *p*<0.05). Relative to the rest of the sample, being employed was associated with lower odds of suicide attempt (OR=0.60, *p*<0.05). Respondents who perceived above-average discrimination had over three times the odds of attempted suicide (OR=3.57, *p*<0.001).

For both transgender and nontransgender respondents, a high level of identity acceptance was associated with fewer depression symptoms (Table 4). One notable difference between the two groups, however, lies in how identity acceptance was associated with depression symptoms. Among nontransgender respondents, 42.2% of the respondents with low self-acceptance of LGBT identity reported more depression symptoms compared with 71.4% among transgender respondents. This was also confirmed by the adjusted odds ratios after controlling for the effect of selected explanatory variables. Among transgender respondents, relative to those with a low level of self-acceptance of LGBT identity, the adjusted odds for respondents with a high level of self-acceptance to report more depression symptoms were 0.04 (*p*<0.001) compared with 0.53 (*p*=0.002) among nontransgender respondents.

**Table 4.** **Self-Acceptance of LGBT Identity and Depression Symptoms in the Past Week Among Transgender and Nontransgender Respondents**

	Transgender			Nontransgender		
Self-Acceptance Level	Not depressed	Depressed	Total	Not depressed	Depressed	Total
Low (%)	14 (28.6)	35 (71.4)	49 (100)	174 (57.8)	127 (42.2)	301 (100%)
High (%)	28 (66.7)	14 (33.3)	42 (100)	272 (73.7)	97 (26.3)	369 (100%)
Chi-square	13.2			18.8		
*p*	<0.001			<0.001		
Adjusted odds ratios on depression symptoms in the past week^a^						
Low	1.00 (reference)			1.00 (reference)		
High	0.04^b^ (0.01, 0.25)			0.53^c^ (0.36, 0.79)		
Number of cases	78			591		
Percentage predicated correctly by the model	80.8			74.1		
Cox and Snell R Square	0.46			0.20		

^a^ The odds ratios associated with self-acceptance of LGBT identity were adjusted for the effect age, race, rural residence, marital status, education, income, employment status, self-rated health, HIV status, daily smoking status, use of illicit drugs, and reported discrimination.

^b^ *p*<0.001.

^c^ *p*<0.01.

## Discussion

Mental health is an essential dimension of overall health, and mental disorders represent one of the leading causes of premature death and disability across the globe.^32,33^ Although the LGBT population makes up a small percentage of the overall population, the mental health needs of those who identify as gender and sexual minorities are substantial.^2–10^ Current research on LGBT mental health is rather limited with even less research focused on transgender individuals.^34^ An important barrier is that for now there has been no systematic collection of data on sexual orientation and gender identity at the national level. For example, of the 50 states participating in the Behavioral Odds Factor Surveillance System (BRFSS), only 27 states have, on their own initiative, begun asking questions about sexual orientation, and even fewer states have started to ask questions on gender identity.^35^

Based on survey data collected from respondents who self-identified as sexual or gender minorities in Nebraska, the present study compared transgender and nontransgender respondents in terms of reported discrimination, depression symptoms, and suicide attempts. Relative to nontransgender respondents, transgender individuals were at higher odds of reporting discrimination, depression symptoms, and attempted suicides. Moreover, the elevated odds associated with transgender identity were robust after controlling for selected variables in demographics, SES, health behavior, self-rated health, and identity acceptance.

The high prevalence of depression symptoms and attempted suicides among transgender respondents in the study could be related to the alarming rates of exposure to discrimination in this population. Based on analysis of suicide attempt and its predictors among 515 transgender individuals, one study reported that gender-based discrimination and gender-based victimization were independently associated with attempted suicide.^18^ Discrimination and prejudice against transgender people are pervasive. Recent evidence from Virginia suggests that transgender Virginians experienced widespread discrimination in healthcare, employment, and housing.^19^ Over 40% of transgender respondents in our study reported a high level of discrimination, pointing to a need to identify the sources of discrimination and address the issue through legislation and public education. Hate crime legislation is needed to increase protection of transgender individuals from discrimination, rejection, and violence.^21,36^ In addition, interventions to increase public awareness of mental health disparities related to transgender persons should emphasize the importance of changing community attitudes to foster greater acceptance. State-level structural stigma was associated with lifetime suicide attempts among transgender adults based on recent findings from a nationwide sample.^37^

Findings from this study reinforce the urgency of suicide prevention among transgender individuals. Among transgender respondents, 37.7% reported attempted suicide compared with 15.9% among the rest of the sample. This is consistent with corresponding findings from previous studies where a substantial percentage of transgender persons reported suicide ideation.^15,16,18^ Certain groups of transgender persons may be even more vulnerable to suicide ideation, including those on the female-to-male spectrum, those with a history of psychiatric hospitalizations, and those who experienced transgender-related violence.^38^ Limited access to competent healthcare services is a critical barrier to suicide prevention among transgender persons, and the economic disadvantage disproportionately experienced by transgender individuals can further restrict timely access to needed care. Furthermore, there is a gap between the healthcare needs of transgender individuals and the supply of care providers who have the sensitivities and expertise to provide culturally relevant care.^39^

One interesting finding from this study concerns the association between identity acceptance and reduced depression symptoms. Even after controlling for selected variables on transgender identity, demographics, SES, self-rated health, and health behavior, respondents who indicated a higher level of identity acceptance were substantially less likely to report depression symptoms. Furthermore, our study provides preliminary evidence that lack of identity acceptance might pose more harm to mental health among transgender individuals than among nontransgender individuals. Depression symptoms among sexual and gender minorities, including transgender persons, may relate to their own perception of gender identity and/or sexual orientation. Exposure to stigma, prejudice, and discrimination could lead to questioning, rejection, and internalized shame of their own sexuality, constituting a significant source of depression symptoms.^40^ Programs aiming to improve identity acceptance among transgender persons could potentially help these individuals better cope with experiences of stigma and discrimination and thus reduce the odds of depression symptoms.

## Limitations

Several limitations of the study are noteworthy. First, while the use of an online survey provides convenience to respondents and helps lower the cost of the study, it precludes those sexual and gender minority individuals who do not have access to computers or the internet. Since the recruitment effort, including study advisement, was primarily focused in the Omaha metropolitan area,^28^ LGBT individuals who live in the rural areas of Nebraska might have been less represented in our sample. Second, key measurements used in the study such as reported discrimination, depression symptoms, and attempted suicides were all based on self-report data. As a result, recall bias might be an issue in some of the data we used. Finally, of the 767 subjects in the survey, only 91 identified as transgender. These respondents also included those who self-identified as gender-nonconforming LGB individuals. The limited sample size prevented us from conducting some further analysis such as to examine how specific transgender types (e.g., female–male vs. male–female transitions) might be related to the mental health outcomes discussed in the study.

Despite these limitations, this study represents a rare effort in assessing mental health outcomes among transgender persons in a relatively conservative Midwestern state. The findings indicate that relative to nontransgender LGB individuals, transgender individuals were more likely to report discrimination, depression symptoms, and attempted suicides. Much of the existing health disparities research concerning transgender individuals has been based on comparisons between transgender and cisgender individuals broadly and not within the LGBT community.^41,42^ Our findings show that even within the already marginalized LGBT community, transgender individuals have worse mental health relative to their cisgender LGB peers.

## Conclusions

Transgender identity was associated with higher odds of reported discrimination, depression symptoms, and attempted suicides. There was a positive association between reported discrimination and depression symptoms among the LGBT population in Nebraska. Self-acceptance of LGBT identity was associated with fewer depression symptoms and this was especially the case among transgender individuals. Protective legislation and public education on gender identity can potentially reduce discrimination against transgender individuals, which is a needed step forward to create a social environment that fosters understanding and respect across social and cultural groups, irrespective of gender identity and sexual orientation.

## Abbreviations Used

BRFSS: Behavioral Odds Factor Surveillance System
CBPR: community-based participatory research
CES-D: Centers for Epidemiological Studies–Depression Scale
NTDS: National Transgender Discrimination Survey
